# Systematic evaluation of intakes in patients receiving non invasive ventilation. the starve study

**DOI:** 10.1186/2197-425X-3-S1-A827

**Published:** 2015-10-01

**Authors:** M Arnaout, A Marincamp, M Reffiena, A-S Debue, M Le Bras, C Boulila, P Lucas, S Cabon, A Feltry, F Daviaud, J Charpentier, J-P Mira, A Cariou, J-D Chiche, Groupe de Travail sur la Ventilation

**Affiliations:** Cochin Port Royal, MICU, Paris Cedex 14, France

## Introduction

Noninvasive ventilation (NIV) is increasingly used in ICUs. Whereas there are specific guidelines related to feeding of critically ill pts receiving invasive ventilatory support, there are no guidelines related to feeding pts treated with NIV. We hypothesize that pts may be significantly underfed during the first 5 days of ventilatory support with NIV.

## Objectives

We conducted this study to evaluate caloric intakes of pts receiving NIV irrespective of the indication for NIV.

## Methods

Prospective, multidisciplinary, observational study in a 24-bed MICU. Inclusion criteria: all pts treated with NIV for more than 4H/day. Exclusion criteria: pts receiving parenteral or enteral nutrition in the management of underlying diseases or comorbidities. Data collected include demographics, pts medical characteristics, NIV indication & duration of NIV sessions, complications developed in the ICU, and nutritional data. We prospectively quantified and recorded all calories intakes through oral, enteral and parenteral routes during the whole duration of the ICU. We also measured BMI and albumin concentrations at the beginning/end of the ICU stay. Data are presented as median [IQR].

## Results

90 pts (45M/45F, 73 y.o. [64,91], SAPS2 38 [33,48], SOFA 4 [2, 6.], IBW 77 [63,94]) have been enrolled in the study. ICU length of stay was 4 [6,9] days and mortality 8.9%. Indication (classified according to the SRLF/SFAR consensus conference^1^) were Group 1 (n = 52), Group 2+ (n = 8) or Group 2- (n = 30). Main results are summarized below. The majority of pts treated with NIV only received less than 1000 Cal/day.

## Conclusions

Pts treated with NIV for more than 4H/days have limited oral intakes and are significantly underfed. Long sessions of NIV, fear of aspiration, and underestimation of the patient severity may explain the failure to provide adequate nutritional support in these pts. A multicentre study is underway to assess feeding practices in NIV pts.

**Table 1:** Table 1

	Day 1	Day 2	Day 3	Day 4	Day 5
**Duration of NIV (h)**	7 [4,10.5]	9.2 [5,14.7]	9.2 [4.5,11.5]	7.8 [4,11]	6.6 [4,10.7]
**Oral intakes (cal)**	123 [0,472]	510 [112,837]	675 [340,1032]	700 [225,1285]	550 [350,800]
**Parenteral nutrion (cal)**	67 [27,126]	91 [45,209]	109 [49,290]	168 [86,343]	122 [60,478]
**NIV pts with <1000 cal/d (n)**	53/58	45/58	41/58	42/58	45/58

*[NIV sessions & intakes]*
